# Understanding the Cellular Origin of the Mononuclear Phagocyte System Sheds Light on the Myeloid Postulate of Immune Paralysis in Sepsis

**DOI:** 10.3389/fimmu.2018.00823

**Published:** 2018-04-24

**Authors:** Lionel Franz Poulin, Corentin Lasseaux, Mathias Chamaillard

**Affiliations:** Univ. Lille, CNRS, INSERM, CHU Lille, Institut Pasteur de Lille, U1019 – UMR 8204 – CIIL – Center for Infection and Immunity of Lille, Lille, France

**Keywords:** dendritic cell, monocytes, ontogeny, sepsis, endotoxemia

## Abstract

Sepsis, in essence, is a serious clinical condition that can subsequently result in death as a consequence of a systemic inflammatory response syndrome including febrile leukopenia, hypotension, and multiple organ failures. To date, such life-threatening organ dysfunction remains one of the leading causes of death in intensive care units, with an increasing incidence rate worldwide and particularly within the rapidly growing senior population. While most of the clinical trials are aimed at dampening the overwhelming immune response to infection that spreads through the bloodstream, based on several human immunological investigations, it is now widely accepted that susceptibility to nosocomial infections and long-term sepsis mortality involves an immunosuppressive phase that is characterized by a decrease in some subsets of dendritic cells (DCs). Only recently substantial advances have been made in terms of the origin of the mononuclear phagocyte system that is now likely to allow for a better understanding of how the paralysis of DCs leads to sepsis-related death. Indeed, the unifying view of each subset of DCs has already improved our understanding of the pivotal pathways that contribute to the shift in commitment of their progenitors that originate from the bone marrow. It is quite plausible that this anomaly in sepsis may occur at the single level of DC-committed precursors, and elucidating the immunological basis for such a derangement during the ontogeny of each subset of DCs is now of particular importance for restoring an adequate cell fate decision to their vulnerable progenitors. Last but not least, it provides a direct perspective on the development of sophisticated myelopoiesis-based strategies that are currently being considered for the treatment of immunosenescence within different tissue microenvironments, such as the kidney and the spleen.

## Introduction

### Where Do We Stand in Regard to the Ontogeny of Dendritic Cells?

The mononuclear phagocyte system has been initially formulated by the late 1960. It consists of a network of cells, comprising monocytes, macrophages, and dendritic cells (DCs) that are dis-seminated throughout the organism. These cells are characterized by their morphology, their phenotypic characteristics (including phagocytic activity), and their roles in orchestrating the immune system. The majority of their committed progenitors are quiescent at homeostasis, although their very high proliferative potential provides them with the capacity to continuously maintain their numbers. Significant progresses in system biology have been made only recently in regard to understanding of the ontogeny and the function of mononucleated cells (referred to as myelopoiesis). This led to the discovery of committed precursors for adult-derived monocytes, conventional, plasmacytoid, or monocyte-derived dendritic cells (Mo-DCs), which are primarily described in the present perspective article. For more details on the embryonically derived phagocytes, we direct the reader to the following outstanding review (1).

Macrophage and DC precursor cells (referred to as MDP) does not constitute a homogeneous population but rather consists in a mixture of progenitors committed either to the DC lineage or the monocyte/macrophage lineage when they are transferred into the bone marrow (BM) of hosts that have previously been irradiated (2, 3). While less is known about the ontogeny of monocytes, macrophages, and DCs in humans than in mice, recent studies have allowed a link to be made with what has been observed in animal models. Notably, a homolog of murine MDP has been identified based on the *in vitro* differentiation of human CD34^+^ hematopoietic progenitors into type 1 conventional DC (cDC1) (4). There has since been a concerted effort to identify precursors restricted to either cDCs or those derived from the monocytic lineage. MDP express M-CSF-R (or CD115) and the Flt3 receptor (CD135), which are receptors for cytokines that play important roles in the development of monocytes or DCs, respectively. It is likely that the commitment shift of MDP depends on the balance between signals linked to the activation of these receptors (5). This hypothesis is bolstered by the fact that the expression of M-CSF-R decreases in the precursors of cDCs and plasmacytoid DCs (pDCs), although it is not detectable in mature cells. Conversely, Flt3 is not found in the precursors restricted to the monocytic lineage (6, 7). Signaling by the aforementioned growth factors could induce changes at the level of the expression of certain transcription factors. For example, the hematopoietic transcription factors PU.1 and MAFB (for MAF BZIP Transcription Factor B) are crucial for the development of DCs or monocytes, respectively, and they could be implicated in engagement in one of these lineages (8).

**Figure 1:**
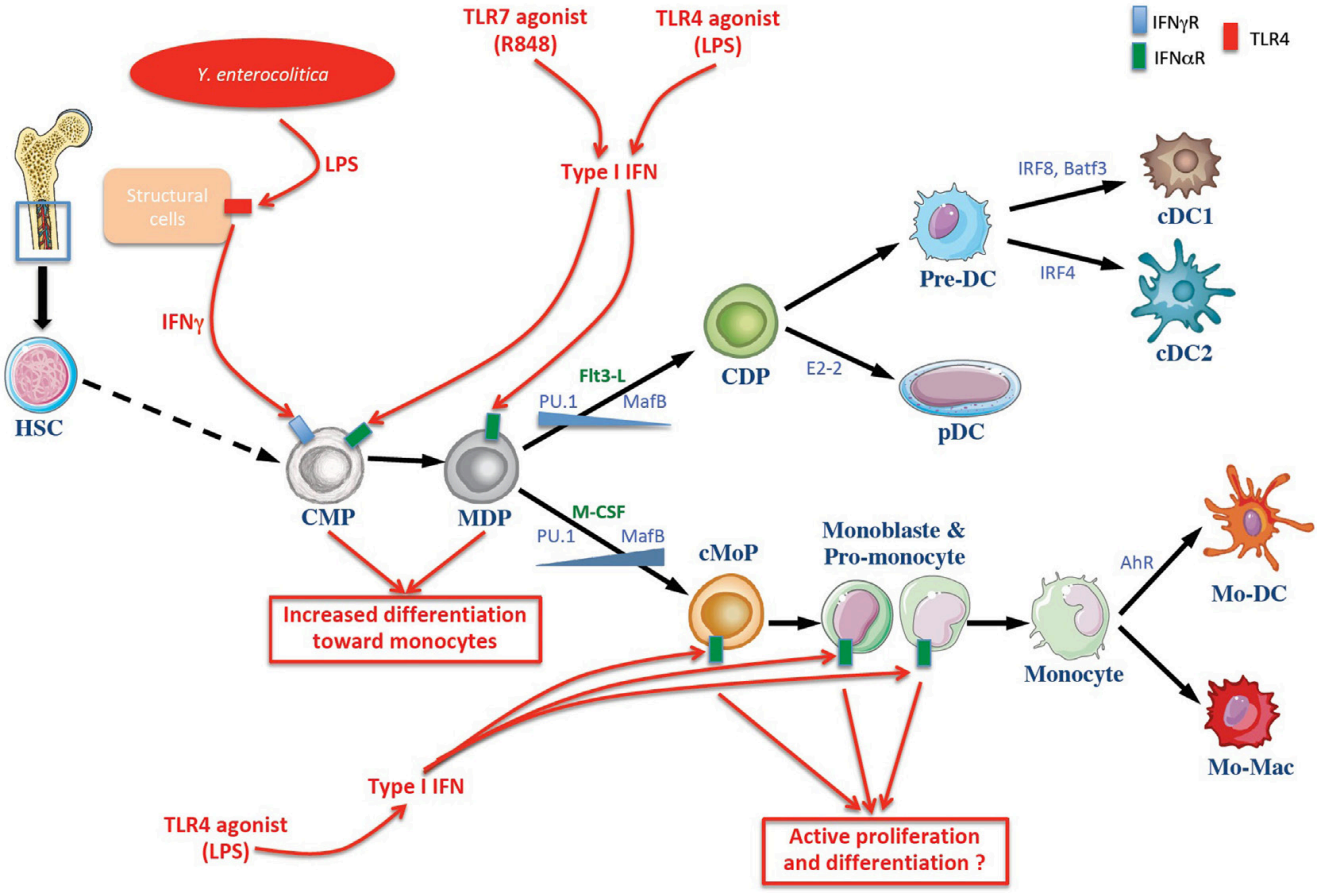
Schematic overview of dendritic cell (DC) and monocytes generation at homeostasis and in systemic infection or endotexemia murine models. The common myeloid progenitor (CMP) derived from hematopoietic stem cells (HSCs) in the bone marrow and can give rise to the monocyte and DC progenitor (MDP) which in turn differentiates into the DC or monocytic lineages. The differentiation toward DC and monocytes is influenced by cytokines and growth factors (noted in green), notably Flt3-L and M-CSF. Transcription factors involved in cell’s fate choice are noted in blue. Infectious stimuli (in red) can affect this process. Lipopolysaccharides (LPS) of the Gram negative bacilli *Yersinia enterocolitica* are sensed by radio-resistant cells that produce IFNγ, inducing a selective differentiation of myeloid progenitors toward the monocytic lineage (monocytopoiesis) at the expense of conventional DC (cDC) (17). Moreover, R848 and LPS induce the production of type I IFN involved in the differentiation of myeloid progenitors toward the monocytic lineage (18, 19). cDC, conventional dendritic cell; CDP, common dendritic cell progenitor; Pre-DC, precursor of cDCs; pDC, plasmacytoide DC; cMoP, common monocyte progenitor; Mo-DC, monocyte-derived dendritic cells, Mo-Mac, monocyte-derived macrophages; IFNγ, interferon γ; TLR toll-like receptor.

Apart from the MDP, the precursor CDP stands for common DC progenitor (Figure 1). Like the MDP, it expresses M-CSF-R and Flt3 (9–11). The CDP on the one hand generates pDCs, and on the other hand generates pre-cDCs, which are the direct circulating precursors of the cDCs in tissues. In parallel, other teams have elegantly shown that, as is the case with mice, the generation of cDC1 and cDC2 by common DC progenitor (hCDP) occurs by production of a circulating progenitor, namely the hPre-cDC, which is incapable of generating pDCs (12). Like their murine homologs, hPre-cDCs are heterogeneous and they comprise various fractions already committed to become cDC1 or cDC2 (13–15). Pre-cDCs leave the BM via blood circulation and then penetrate into lymphoid and non-lymphoid tissues in order to differentiate into cDCs (9–11). The factors that influence the differentiation of pre-cDCs into cDC1 or DC2 are still unknown. However, it appears that this decision is taken at the CDP stage, which can already exhibit a transcriptional signature similar to cDC1 or cDC2. Moreover, the pre-cDC population appears to be heterogeneous, comprising a mixture of pre-cDC1 and pre-cDC2 in mice (16) and in humans (15).

More recently, a progenitor restricted to monocytes and derived directly from MDP was identified and designated as cMoP, for common monocyte progenitor (Figure 1). It differs phenotypically from MDP by the loss of Flt3 expression. Consequently, cMoPs differentiate into monocytes and their descendants, but they do not generate cDCs (7). The development of cMoPs into monocytes also takes place as monoblast and then as pro-monocyte stages. They are characterized by the expression of stem cell antigen 1 (Sca-1) and they undergo very fast turn-over in the BM (20, 21). The monocytes generated in this manner then migrate from the BM to the tissues where they differentiate depending on the microenvironment (22). Furthermore, a recent study has shown that the generation of human monocytes by hMDP occurs by production of restricted precursors referred to as cMoPs (23), as in mice (7).

These novel concepts are not yet set in stone, however, as the single cell genomic era is already leading to refinements in ontogeny of each subsets of DCs and macrophages. For instance, Helft and colleagues recently demonstrated that human cDC1 are derived more efficiently from the multipotent lymphoid progenitor than from the common myeloid progenitor (CMP) (24). In parallel, it is proposed that the MDP does not constitute a homogeneous population but rather consists in a mixture of progenitors committed either to the DC lineage or the monocyte/macrophage lineage, with no or only very few individual cells able to yield both cell lineages in their progeny (25, 26).

### The Myeloid Enigma of Sepsis-Related Mortality

Monocytopoiesis is a dynamic process that occurs in the BM as well as in other organs as an adaption to several physiological stresses that varies over time, while emergency myelopoiesis refers to the rapid generation of myeloid effector cells in response to purified lipopolysaccharide (LPS) (27). A hallmark of septicemia is a profound decrease of circulating DCs, which is also an indicator of a poor prognosis for septic patients (28–30). Two main types of murine models of sepsis or acute inflammation are generally used. On the one hand, the model of peritoneal or intravenous injection of purified endotoxins constitutes a simple model of acute inflammation. On the other hand, the other widely used murine sepsis model is based on cecal ligature and puncture (CLP). This chirurgical model induces intestinal bacterial translocation into the peritoneal cavity, generating a systemic infection and massive inflammation. Meanwhile, the extent to which murine models adequately reflect the complexity of human sepsis or acute inflammation is a matter of debate (31, 32). Although differences in TLR distribution among the various mononuclear phagocyte subsets exist between humans and mice (33), the latter are widely used to understand part of these complex disorders.

In order for this emergency myelopoiesis to be induced, TLR4 needs to be expressed by the radiation-resistant cells of the host. These cells then produce the growth factor G-CSF, which is sufficient to induce this phenomenon (34). G-CSF can also be produced following activation of inflammasomes, which depends on the cytokines IL-1beta and IL-1alpha, thereby inducing emergency myelopoiesis (35, 36). A recent study has also provided evidence for the production of IL-3 by B lymphocytes in a murine model of septicemia. This IL-3 allows for a significant increase in the production of monocytes and neutrophils, which are involved in systemic inflammatory respiratory syndrome (SIRS) and the “cytokine storm.” Furthermore, an elevated level of IL-3 in serum is predictive of a poor prognosis in septic patients (37). The hematopoietic progenitors can hence be indirectly activated in case of severe infection, so as to reorient the production of cells toward the myeloid lineage. However, the mechanisms causing this decrease in DCs during sepsis remain unclear, possibly encompassing both enhanced cell death of at least some subsets of DCs (and their defective reconstitution from their progenitor cells, e.g., originated from cMop and/or pre-cDC). Using the recently accepted nomenclature (38), we herein discuss the potential mechanisms causing this decrease in some DCs during sepsis that is linked to long-term sepsis-related mortality, especially in elderly and diabetic populations. In addition to studies of DCs in sepsis and endotoxemia models, we also review the contribution of macrophages, as these phagocytes have sometimes been incorrectly classified as Mo-DCs with the use of non-discriminating markers (38), such as CD64.

#### Monocytes

Monocytes are circulating hematopoietic cells generated in the BM, with a very short half-life that does not exceed a few days. In mice, they are generally divided into two subpopulations that are distinguished based on the expression of Ly6C surface molecules (39). Ly6C^hi^ monocytes, or inflammatory monocytes, are rapidly recruited at sites of infection and inflammation in a CCR2 chemokine-dependent manner. Once inside tissues, diverse signals from the microenvironment can induce an increase in phagocytosis, the production of cytokines, antimicrobial activity, and antigen presentation by the cells, thereby inducing a phenotype that is sometimes very similar to that of macrophages or DCs (22, 40). Ly6C^low^ monocytes, also called patrolling monocytes, are less common than inflammatory monocytes, and they express the CX3C chemokine receptor 1 (CX3CR1) also known as the G-protein coupled receptor 13 (GPR13) or fractalkine receptor. Indeed it appears that their main function is to ensure endothelial integrity, by patrolling in the lumen of the blood vessels along the endothelium (41). These cells are the product of the differentiation of Ly6C^hi^ blood monocytes at homeostasis (42). Ly6C^low^ monocytes could hence be considered as macrophages of the vascular system (22, 40). Two monocyte populations are also present in humans, and they correlate with those found in mice (43, 44). However, they are not distinguished based on the same surface markers as in mice. Rather, they are distinguished by the expression of the LPS CD14 coreceptor and of the CD16 receptor for crystalizable fragments of antibody. Human CD14^+^CD16^−^ monocytes appear to be the homologs of the murine Ly6C^hi^ population, while the CD14^+^CD16^+^ population appears to be analogous to the murine Ly6C^low^ population (22, 40, 41). During polymicrobial sepsis, inflammatory monocytes prevent renal damage in a CX3CR1-dependent adhesion mechanism (45) and a decrease in circulating patrolling monocytes is associated with unfavorable outcome (46). While reactivity to the subsequent endotoxin challenge is enhanced by muramyldipedtide, it remains to be determined whether the anaphylactic reactions are influenced by the muramyldipeptide-induced conversion of Ly6C^hi^ toward Ly6C^low^ monocytes (47) or by other mechanisms involved in leukocyte binding and adhesion.

#### Macrophages

Adult-derived macrophages have been assumed to be the progeny of monocytes in tissues (48). However, although monocytes can indeed generate macrophages under certain conditions, circulating monocytes do not appear to be the main source of these cells. With the aim of simplifying nomenclatures, Martin Guilliams and his collaborators recently proposed that the use of the term “macrophage” should be restricted to mononucleated phagocytes of embryonic origin (49). Indeed, recent studies have shown that the majority of macrophages residing in the brain, the liver, the lungs, and even the spleen are derived from embryonic precursors in the vitellin vesicle and in the fetal liver. These macrophages disseminate to the various tissues of the body once the blood circulation becomes established, and they are maintained there by proliferating locally throughout the individual’s lifetime (42, 50–57). These embryonic macrophages can be progressively displaced by blood-derived monocytes. For instance, the intestinal macrophages that are of embryonic origin are replaced by the differentiation of blood monocytes that are recruited into tissues several weeks after birth (58). Moreover, monocytes constitute a major source of tissue macrophages already at steady state, including the skin (59) and the oral mucosa (60), and this phenomenon is amplified by inflammatory and/or aging processes in a range of organs such as the intestine (61), the heart (62, 63), the peritoneal cavity (64), and the liver (65, 66). Hotchkiss et al. reported that the number of splenic macrophages is not reduced in septic and trauma patients (67). These observations still need to be investigated with up-to-date markers to decipher the exact changes in the mononuclear phagocytes at the subset level. Indeed, the identification of splenic macrophages with CD14 is not sufficient.

#### Monocyte-Derived Antigen-Presenting cells

Upon homeostasis in certain tissues such as the kidney, or in case of either infection or inflammation, numerous studies have shown that some DCs and macrophages are two sides of the same coin, as they both are derived from monocytes. These cells will hence be referred to here as monocyte-derived antigen-presenting cells (Mo-APCs). The cells derived from monocytes can express high levels of major histocompatibility complex class II (MHC-II) and CD11c, and they can migrate and efficiently present Ag to T lymphocytes (59). Certain studies have also shown their efficacy at cross-presentation of Ag, although these cells appear to use different intracellular components than cDC1 to achieve this (68–71). As suggested by their cross-presenting activity, like cDC1, APCs derived from monocytes have been implicated in cytotoxic Th1 responses (72, 73). However, like cDC2, Mo-APCs have also been reported to induce Th2 and Th17 types of responses (74–76). Depending on the context, Mo-APCs could develop functions similar to those of the various populations of cDCs. However, the lack of markers to discriminate these cells from cDCs, macrophages, or active monocytes greatly complicates the study of Mo-APCs. A study has shown the presence of MHC-II^+^CD11c^+^ cells derived from monocytes in skeletal muscles under conditions of homeostasis. The intramuscular administration of alum adjuvant induced a very pronounced increase in the representation of these cells and the simultaneous administration of LPS greatly increased their capacity to migrate to lymph nodes and the spleen. These cells are capable of presenting Ag to naive T lymphocyte by normal as well as cross-presentation. They are characterized by the expression of inducible nitric oxide synthase (iNOS) and of the Fc receptor CD64 (FcγRI), which are not expressed by cDCs and pDCs (69). The CD64 marker can also be used to distinguish CDP-derived cells from monocyte-derived cells at the level of the intestine and the skin under homeostatic or inflammatory conditions in mice (59, 61), but not at the level of the kidney (77). The immunoglobulin FcεRI receptor has also recently been reported to be expressed by Mo-APCs in mice and in humans (70, 74), and on human cDC2 (15, 78, 79), it appears that Fc receptors are mainly restricted to phagocytes of monocytic origin (80), thereby they may facilitate their identification in conjunction with other discriminative traits (80). Improvements in the characterization of these cells within distinct tissue microenvironments will undoubtedly increase our knowledge of their biology and presumably provide an explanation for the poor clinical impact of past investigations regarding each DC subsets in sepsis. Despite these limitations, Kassianos et al. demonstrated that human Mo-APCs are the major subsets responsive to *Escherichia coli* in terms of inflammatory cytokine secretion, antigen presentation to CD8^+^ T cells, and phagocytosis (33).

#### Dendritic Cells

Dendritic cells are the main antigen-presenting cells (APCs) of the organism. They are characterized by the expression of MHC-II, integrin CD11c, and the transcription factor Zbtb46 (sometimes referred to as zDC) (81–83). However, these markers are also expressed by certain macrophages or other cells that are derived from monocytes. Generally, a distinction is made between cDCs, plasmacytoid (pDCs), which are present in the basal state, and DCs derived from monocytes (Mo-DCs), which are recruited extensively in case of inflammation (6). cDCs are found in the vast majority of lymphoid and non-lymphoid tissues. The term “conventional” refers to DCs that are non-plasmacytoid and that are not derived from monocytes but from a precursor restricted to these cells. cDCs induce either immunity or tolerance toward the Ag that they present to lymphocytes (6). There is a general consensus that there are two populations of cDCs, namely cDC1 and cDC2, which are endowed with distinct functional specialization and thus play complementary roles in the shaping of immune responses (6, 49, 83, 84). Numerous studies have shown a dramatic decrease in DCs during septicemia. Integrin CD11c is often used as a marker of DCs, although it is also expressed to a varying degree by other cell populations such as certain macrophages, neutrophils, and lymphocytes (Table 1). A dramatic decrease in CD11c^+^ cells in the periphery has been observed over the first days of a murine model of polymicrobial sepsis (85–90), and in the BM (91) (Table 2). Wen and colleagues found that there was a significant reduction in the percentage of CD11c^+^CD11b^+^MHCII^hi^ cells in lung and spleen from 3 to 14 days post-CLP procedure in comparison to sham mice (86). They also noted a decrease in the percentage of lung CD11c^+^CD11b^+^ and CD11c^+^B220^+^ cells at days 2 and 8 in post-CLP mice infected by *S. mansoni* eggs, which was associated with a diminished ability of lung CD11c^+^ cells to produce IL12p70 after TLR agonist stimulation (88).

**Table 1:** Different markers used to distinguishe mouse and human phagocytes.

Subset	Phenotype		Reference
	Mouse (spleen)	Human (blood or ascites)	
cDC1	MHC-II^hi^ CD11c^hi^ Clec9^+^ XCR1^+^ CD8^+^ CD4^−^ CD24^+^ CD64^−^	HLA-DR^+^ CD11c^+^ Clec9^+^ XCR1^+^ CD141^+^ CD14^−^ CD16^−^	(4, 7, 100–103)

cDC2	MHC-ll^hi^ CD11c^hi^ CD11b?^+^ SIRPα^+^ CD8^−^ CD4^+^ CD64^−^	HLA-DR^+^ CD11c^+^ CD11b^+^ CD1a^+^ CD1c^+^ CD14^−^ CD16^−^	(4, 72, 102, 103)

pDC	CD11c^int^ SiglecH^+^ B220^+^	HLA-DR^+^ CD123^+^ CD303^+^ CD11c^−^ Axl^−^ CD2^−^	(4, 11, 102, 104, 105)

Mo-DC	MHC-II^+^ CD11c^+^CCR2^+^ CD64^+^	HLA-DR^+^ CD11c^+^ CD1a^+^ CD1c^−^ CD141^−^	(53, 63, 102, 103, 106, 107)

Mo-Mac	CD64^+^ F4/80^+^ MERTK^+^	HLA-DR^±^ CD16^+^	(53, 63, 103, 106, 107)

**Table 2:** Comparison of cell numbers for mononuclear phagocytes populations in murine models of polymicrobial sepsis, systemic inflammation, or endotoxemia.

Reference	Model	Organ	pDC	Total CD11c^+^ cells	Monocytes and derivatives	cDC1-like	cDC2-like	DN
(85)	CLP	Spleen, peritoneum	?	➘	?	?	?	?
(92)	CLP	Lymph nodes	?	➘	?	?	?	?
(84)	CLP	Spleen	?	➘	?	?	?	?
(93)	CLP	Lung	?	➘	?	?	?	?
(88)	CLP	Lung	➘	?	?	?	➘	?
(86)	CLP	Spleen, lung	?	➘	?	?	?	?
(94)	CLP	Spleen	?	➘	?	➘	➘	✘
(91)	CLP	Spleen, bone marrow	?	➘	?	➘	➘	✘
(89)	CLP	Spleen	?	➘	?	➘	➘	➚
(95)	CLP	Spleen	?	➘	?	➘	➘	✘
	LPS injection		?	➘	?	✘	➘	➚
	P3CSK4 injection		?	➘	?	➚	➘	➚
(96)	*Escherichia coli* infection	Spleen	?	➘	?	➘	➘	➘
	LPS injection		?	➘	?	➘	➘	➘
(97)	*Yersinia enterocolitica* infection	Spleen	?	?	➚	?	?	?
(98)	LPS injection	Spleen	?	?	?	➘	✘	?
(99)	LPS injection	Spleen	?	?	➚	➘	➘	?

*Down or up arrows mean that the cell number is decreased or increased compared to controls, respectively. The cross means that the cell number is not significantly changed compared to controls. Cell numbers described here were measured within 24 h after injection for endotoxemia models or within a week after infection or surgery for CLP and systemic infection models. cDC1-like cells correspond to CD8^+^ or CD11b^−^ DCs. cDC2-like cells correspond to CD11b^+^ or CD4^+^ DC. CLP, cecal ligation and puncture; pDC, plasmacytoid dendritic cells; cDC, conventionnal DC; DN, double negative DC for CD4 and CD8 markers; LPS, lipopolysaccharides*.

In order to evaluate the impact of septicemia on different populations of DCs, Flohé and his collaborators used a mouse CLP model, and they distinguished CD11c^+^ cells on the basis of expression of CD8 (expressed by cDC1) and CD4 (expressed by cDC2), from double-negative cells that might be cDC precursors, for example (108). In light of this, the observed loss of DCs at 36 h postoperative to the procedure was due to the CD8^+^ and CD4^+^ populations, while the total number of double-negative cells itself was increased in this model (94). Another CLP study has also provided evidence for a depletion of DCs from the local mesenteric and systemic inguinal lymph nodes, with a preferential loss of cDC1 expressing CD8, which was associated with increased apoptosis (92). This splenic cDC1 loss was maintained up to 5 days post-CLP procedure, and the cells repopulated the spleen at day-7 post-CLP procedure in an NF-kB signaling-dependent pathway (90).

It hence appears that, in mice, polymicrobial septicemia induces a specific depletion of certain DC populations of the lymphoid organs. Some populations may be depleted more so than others, as has been observed in the spleen with an increase in the CD4^–^ CD8^–^ population, for which the exact link with cDC1 and cDC2 is not known (94, 108). In keeping with a less-mature phenotype of this double-negative population, when cell proliferation was measured, during a CLP procedure, by BrdU incorporation after 4 days, these cells had a significantly higher BrdU content compared to sham control mice (91). The double-negative splenic cells were differently affected according to the model selected. Thus, they were not affected after CLP, although they were significantly increased after LPS or Pam3CSK4 injection (95).

#### Type 1 Conventional DC

Type 1 conventional DCs are characterized by the expression of TLR3 that is required for sensing of viral RNA, and by a greater capacity for secretion of the cytokine IL-12p70 following their activation. This cytokine allows for differentiation of type 1 T helper cells (Th1) implicated in cytotoxic anti-viral and anti-tumor immunity (100, 109, 110), and promotes CD4^+^ T helper cells for CD8^+^ responses (101). This subpopulation of cDCs has also been reported to be efficient in terms of a particular mechanism of antigen presentation that is called “cross-presentation”, which allows these cells that are constitutively resistant to viral infection to acquire exogenous antigens from the infectious agent (111). This cross-presentation process consists of the processing of exogenous antigens into peptides and their loading onto MHC-I molecules so as to be presented to CD8^+^ T cells. This process is called cross-priming if it results in their activation (112, 113). In humans, a very similar population has been reported to be present in the blood and in the spleen, expressing CLEC9A, as do their murine homologs (4, 114–117) (Table 1). Overall, the data in humans suggest that cDC1 excels at cross-presentation of cell-associated antigens (115, 118–122) or of antigens that are delivered to late endosomes/lysosomes (123, 124). However, the data in mice show that other DC subsets are also capable of cross-presentation, provided that they have been properly stimulated (125, 126). Meanwhile, this function could depend on a number of variables such as the type of antigen, its intracellular route of delivery, and the accompanying adjuvant signal sensed by the DCs. cDC1 cells are more effective in regard to this function in specific pathophysiological contexts including viral infections or tumor development/treatment (127–129). During LPS-induced endotoxemia in mice, a reduced cross-priming activity of splenic cDC1 (130) correlates with a prominent loss of splenic cDC1, defined as CD8^+^ DCs, which is glucocorticoid dependent (98). Indeed, endogenous glucocorticoids blunt LPS-induced inflammation and they promote tolerance by suppressing cDC1 IL-12 production. In the absence of glucocorticoid signaling in CD11c-expressing cells, LPS treatment induces higher serum levels of IL-12, type I IFN, TNF-α, and IFN-γ (98). In terms of epigenomic reprogramming, the inflammatory function of TNF is potentiated by type I IFN by prevention of the silencing of genes encoding inflammatory molecules in human macrophages (131). A similar decrease in mouse splenic cDC1 has also been observed after CLP procedures (89, 95). However, injection of different PAMPs induced various effects on cDC numbers. Indeed, LPS does significantly affect splenic cDC1 numbers within 2 days after LPS injection (96), which is followed by a cDC1 number recovery (95, 96) (Table 2). On the other hand, Pam3CSK4 does induce an increase in cDC1 cells after 4 days (95) (Table 2). Like their mouse counterpart (114, 132), human blood cDC1 cells were found to not express or very low level of TLR4 and they failed to ingest *E. coli* (33). It remains to be investigated whether these differences are still maintained in human lymphoid and non-lymphoid tissues during sepsis and endotoxemia.

#### cDC2

By contrast, cDC2 are often characterized by the expression of integrin CD11b and SIRPα (also referred to as CD172a) (80), and in the spleen as CD4^+^CD8^–^ cells (Table 1). They are found in lymphoid and non-lymphoid tissues, and they predominate over the cDC1 population in nearly all tissues. The development and maintenance of cDC2 appear to be dependent, for example, on the IRF4 transcription factor (102, 103, 133) and the activation of Notch-2 receptors (134). This population of cDCs appears to be more efficient than the cDC1 in terms of the interaction with CD4^+^ T lymphocyte and the polarization of helper T lymphocytes, particularly for Th2 and Th17, which are implicated in immune responses toward extracellular pathogens and the regulation of immunity (74, 133, 135–137). However, the heterogeneity of CD11b^+^ cells has greatly complicated the study of this population. Indeed, distinguishing cDC2 from macrophages and cells derived from monocytes is difficult as they can express numerous markers that they have in common. To date, it has hence been difficult to assign them non-overlapping immunological properties as well as to establish their dependence on specific transcription factors. New markers have recently been described to assist with the discrimination between cDC2 and macrophages and the cells derived from monocytes in various tissues, such as the lungs, muscles, and the intestines. A decrease in splenic cDC2 has been observed in CLP models (91, 94, 95), associated with a decrease in proliferation of the residual cells (91) (Table 2). Proper definition of cDC2 cells in various species and tissues, and in various acute inflammatory models and human samples to define their decline in numbers and alteration of their function, still needs to be investigated with the improved definition of the markers (Table 1). Indeed it has been proposed a set of markers to define properly cDC2, and cDC1, across species and tissues (38), and the new refinements by single cell approaches would need to be taken into account to further study cDC2 cell modulations during sepsis and endotoxemia (15, 79, 138).

#### Plasmacytoid DCs

Already in the first papers describing their discovery more than 15 years ago, human and mouse pDCs have been shown to lack any antigen presenting functions at steady state but to acquire it upon proper stimulation. Relative to human pDCs that do not express CD11c, mouse pDCs may express intermediate, not low, levels of this marker. While mouse and human pDCs have been found to express Siglec-H or Blood Dendritic Cell Antigen 2 (BDCA-2) markers, respectively, they are not sufficient to characterize them, since they are also expressed on subsets of macrophages in mouse (16, 139) and of pre-cDCs in human (15, 79). pDCs are found in blood, as well as peripheral lymphoid and non-lymphoid tissues. The main function of pDCs is the rapid and pronounced release of type 1 IFN in case of viral infection, due to activation of TLR7 and TLR9 by viral nucleic acids (140). Despite their role in secreting type I IFNs during endotoxemia, pDCs may also be critically involved in regulating endotoxemia through their function in cross-priming and cross-presentation of antigen to T cells (141–143). While the inability to present antigens of steady state human pDC is widely accepted since their discoveries, measuring this function necessitates both to properly purify pDC ensuring lack of contamination by other DCs (15, 79) and also to segregate pDC according to their different activation states that may be linked to distinct functional specialization (138, 144). Indeed, a proper preparation of pDC from human blood can be reached by studying Lin^−^ (by using these markers: CD14, CD16, CD19, CD20 and CD56), CD123^+^HLA-DR^+^AXL^−^CD11c^−^ cells (Table 1) (15, 79, 138, 144). As few studies have investigated pDC during endotoxemia and sepsis (Table 2) (88), there is a need to revisit the role of pDCs during endotoxemia and sepsis. In the future, these refinements to ensure proper purification of pDCs should allow for a better delineation of the roles of these various populations in immunological processes, such as sepsis (61, 69, 74, 102, 132).

### Toward the Identification of Novel Myelopoiesis-Based Therapeutic Targets in Sepsis

The different types of mononuclear phagocytes might be affected during sepsis by a reduction in their number, by cell death or precursor fate mechanisms, or by their resolutive functions. Numerous studies have shown that immune cell death contributes to immunosuppression and damage to organs during the development of septicemia (145). Apoptosis appears to at least partially explain the loss of DCs observed in a murine model of septicemia (146). For instance, sera from sepsis patients has been shown to induce death of circulating CD11c^+^ CD123^−^ DCs, CD14^+^ monocytes and of *in vitro* generated monocyte-derived DCs (147). However, the markers used do not allow the subset specificity to be determined. Moreover, the potentially lowered survival of pDC needs to be evaluated with more specific markers that preclude pre-DC contamination (15, 79). This programmed cell death is in part due to the engagement of some TLRs (89, 96, 148). For instance, cDC1 apoptosis in the spleen within 48 h following live *E. coli* injection is TLR4- and TRIF-dependent (96). Furthermore, phagocytosis of apoptotic cells by DCs renders them tolerogenic. Immunosuppression induced by an endotoxic shock is restrained by the expression of an anti-apoptotic protein by DCs, or by an increase in their number and their activation state by treatment with Flt3L (106, 107, 149). In conclusion, the increase in apoptotic cells with septicemia could contribute to immunosuppression by, on the one hand, the loss of effector cells, and, on the other hand, the induction of tolerance (150).

In addition to death-mediated modulation of the mononuclear phagocyte system, these cells can also be affected by their functions. For instance, during sepsis, monocytes are reprogrammed to enhance protective functions such as anti-microbial functions, which are dependent on hypoxia inducible factor-1α (151). In contrast to this beneficial modulation, the mononuclear phagocyte system can also be modulated during the course of sepsis to promote immunosuppression. For instance, the mononuclear phagocyte system might lose the ability to drive a suitable adaptive immune response. DCs of septic patients, for example, exhibit a decrease in the expression of HLA-DR, thereby reducing their capacity to interact with T lymphocytes (152). Similarly, DCs of septic mice also exhibit a decrease in the expression of MHC-II (91). Moreover, numerous studies in humans as well as in mice have provided evidence for a pronounced decrease in the production of pro-inflammatory cytokines such as IL-12 or TNF by septic DCs stimulated by several PAMPs, while DC dysfunction during sepsis is partly mimicked by the TLR2 agonist Pam3CSK4, rather than the TLR4 agonist LPS (95). Conversely, their capacity to produce the anti-inflammatory cytokines IL-10 or TGF-β is significantly increased (86, 94, 152, 153). Like DCs, monocytes isolated from the blood of septic patients exhibit decreased expression of HLA-DR molecules and lower production of the pro-inflammatory cytokine IL-12 following stimulation after an increase in the production of the anti-inflammatory cytokine IL-10. A high concentration of IL-10 is particularly associated with a poor prognosis for septic patients (93). In the same way, human blood cDC2 produced immunoregulatory molecules, such as IDO, upregulated PD-L1 (a ligand of the inhibitory co-receptor PD1 on T cells), produced high levels of IL-10, and were immunosuppressive in response to *E. coli* (33). Similarly, PD-1 or PD-L1 was expressed at higher levels in septic shock patients (154), and their functional blockade by antibodies restored monocyte functions (155). In terms of helper T cell (Th) polarization, in mice it appears that the interaction between septic DCs and CD4^+^ T lymphocytes induces preferential polarization of the latter toward a Th2 type or T regulatory profile (86, 91, 153, 156). In addition, GM-CSF-derived BM DCs from CLP- and Pam3CSK4-treated mice were less effective *in vivo* at Th1 priming compared to GM-CSF-derived DCs from LPS-treated mice (95). It is possible that the loss of the capacity to induce Th1 responses is due, at least in part, to a specific loss of cDC1 which appear to be crucial for the development of such responses (92, 94, 107, 112, 113). Although progress has been made in this regard, the exact molecular mechanism of such functional difference remains unclear. Moreover, it has been reported that the failure of DCs generated by post-septic mice to produce IL-12 with the CLP model was observed at least 6 weeks after this process. This failure to produce IL-12 appears to be due to epigenetic changes induced at the level of promoters for genes coding for this cytokine (86). In summary, these long lasting events might occur in myeloid progenitors as DCs are short lived. Future molecular investigations should consider their epigenetic regulation.

In contrast to modulation of the mononuclear phagocyte system at a functional level, sepsis may affect the developmental fate of myeloid progenitor cells. Monocytes can also acquire phenotypic and functional characteristics of DCs, although the factors influencing this differentiation are still unknown. In keeping with the high level of plasticity of monocytes, the differentiation of these cells depends on local mediators such as cytokines, PAMPs, or DAMPs (76, 157). Some of these cytokines are induced by these danger signals, such as type I IFN. Indeed, type I IFN gives rise to Mo-APCs by acting through the IFNAR receptor on direct monocyte progenitors (Figure 1) (18). Similar to DAMPs, microbiota-dependent metabolites affect the balance between Mo-DCs and macrophages. For instance, aryl hydrocarbon receptor (AHR) ligands, derived either from dietary food intake or from tryptophan catabolism at the mucosal barrier, shift the monocyte cell fate toward monocyte-derived DCs in a PRDM1- (also known as BLIMP1) and IRF4-dependent manner (99, 158). It appears that activation of IRF4 allows monocytes to differentiate into Mo-DC while it remains controversial that only monocytes re-expressing Flt3 can generate Mo-APCs (71, 99, 159). It might be of interest to understand the molecular mechanism of how reprogramming of each monocytes impacts their subsequent ability to differentiate into either DCs or macrophages within different microenvironment. In summary, the generation of monocytes at the expense of cDCs could limit the availability of innate immune effector cells that can counter the infection, and this process might be involved in the immunosuppression in septic animals and patients.

Recent studies have shown that hematopoietic progenitors themselves express PRR, such as TLR (160, 161). They can hence theoretically directly detect PAMP and react as a consequence. *In vitro* culture experiments of murine and human HSC stimulated by agonists of TLR have shown their preferential differentiation into phagocytes at the expense of cells of the lymphoid lineage (161–164). Furthermore, experiments with parabiotics have ele-gantly demonstrated that a low number of HSC continuously enter the blood circulation before returning to the BM (165). This phenomenon could allow HSC to locally generate effector cells, directly after encountering a circulating microorganism and in a way that is tailored to the molecular signature of the invading pathogens (166).

Type I IFNs can have an effect on hematopoiesis *in vivo*, particularly by induction of the proliferation of quiescent HSC following injection of the TLR3 agonist polyinosinic: poly cytidylic acid (poly I:C) into mice (167). However, excessive signaling by type 1 IFNs, induced for example by a deficiency in the negative regulator IRF-2, leads to attenuation of HSC proliferation over time, as evidenced by the low capacity of these hematopoietic cells to repopulate following transplantation (168, 169). Additionally, chronic administration of poly I:C induces a selective depletion of WT hematopoietic stem cells (HSCs) in chimeric mice with WT: *Ifnar1*^−/−^ BM cells. Excessive proliferation could, as a matter of fact, induce a state of attenuation of the function of stem cells by differentiation, senescence, or also apoptosis, thereby decreasing the risk of malignant transformation and of perturbation of the tolerogenic tissue architecture (167, 170). This attenuation of stem cells could, over time, lead to leukopenias and hematopoietic anomalies. There is still scant documentation regarding the influence of the infectious context on the potential for differentiation of myeloid precursors. In addition to their roles in regard to HSC, type 1 IFNs are involved in the differentiation of common myeloid precursors into macrophages, following the direct activation of TLR7 of these cells by R848 (19). Also, a study has shown that the precursor cells of common DC progenitors express several TLR in mice, including TLR4. *In vitro* activation of these TLR induces a reduction in the expression of the chemokine receptor CXCR4, which is involved in the retention of CDPs in the BM. *In vitro* activation of the TLR of CDP also induces an increase in the expression of the chemokine receptor CCR7, which is involved in migration of DCs toward the lymphoid organs. When these active CDP were transferred to mice, they were preferentially found at the level of lymph nodes rich in agonists of TLR, subsequent to local TLR agonist injection, where they underwent differentiation. As the various populations of DCs were not studied in detail in this study, it is hence not possible to draw conclusions regarding the potential selective differentiation of stimulated CDP (160). However, a study has shown that the *in vitro* differentiation by the cytokine GM-CSF of hematopoietic cells derived from CLP septic mice induced the generation of DCs with an immunosuppressive phenotype, aggravating the susceptibility to secondary infections with *Pseudomonas aeruginosa* when they were injected into post-CLP septic mice (91, 171). Conversely, the injection of DCs derived *in vitro* from the BM of healthy mice considerably increased the resistance of septic mice to secondary infections (171). Similarly, when injected intratracheally at day-5 after CLP surgery, cultures of DCs isolated from mouse BM with GM-CSF and IL-4 protected recipient mice from *Aspergilus fumigatus*-induced death (153). The exact contribution of each DC subset in cultures of mouse BM with GM-CSF remains to be investigated, as *in vitro* generated CD11c^+^MHC II^+^ cells are a heterogeneous population of cells, with some resembling macrophages more than DCs (172).

Similarly, IL-4 may favor monocyte development toward monocyte-derived DCs to the detriment of monocyte-derived macrophages (99, 172). Aside from a role for IL-4 in the ontogenic shift between DCs and macrophages, IL-4 plays an important role at the functional level as it is required for optimal cross-priming by GM-CSF-induced Mo-DCs (71). A side-by-side comparison of these *in vitro* generated DC subsets in the protection of septic mice needs to be undertaken. Moreover, the contribution of *in vitro* generated cDC1 and cDC2 needs to be investigated by using cultures of mouse BM with Flt3L (173). Finally, supplementation of mouse BM cultures with GM-CSF and IL-4 should be studied so as to determine the contribution of each DCsubset in the resolution of sepsis. As done recently in a cancer model, and because *in vitro* generated DCs might lack environmental cues, the benefit of directly *ex vivo* extracted DC in sepsis models might be of interest (174). Despite this comparison between *in vitro* and *ex vivo* generated DC subsets, their ability to reach the organs of interest, such as lung-draining lymph nodes in the case of intratracheally injected cells, remains to be verified in each sepsis model (153). Meanwhile, limitations of diphtheria toxin-mediated models of cell type depletion have been described such as for DC targeting in CD11c-hDTR mice where many other cell types are affected (175). This implies performing complementation studies with each DC subset obtained *in vitro* or *ex vivo*, for proper interpretation of the phenotype of diphtheria toxin-treated mice. For instance, adoptive transfer of GM-CSF-derived DCs into DC depleted mice prevents CLP-induced mortality (176).

It hence appears that DCs generated during septicemia have different effects compared to those produced under homeostatic conditions, and that they are involved in the immunosuppression observed in septic patients. In various models of bacterial infection, the chemokine CCR2 receptor-dependent mobilization of monocytes is crucial for the control of the pathology. Pasquevich and his collaborators have recently shown that the infection of mice with Gram-negative *Y. enterocolitica* bacilli induces a selective differentiation of myeloid progenitors toward the monocytic lineage (monocytopoiesis) at the expense of cDCs. This process depends on the activation of TLR4 and on the production and the detection of IFN-γ by non-hematopoietic cells (17).

## Concluding Remarks

The word “Septicemia” is of Greek origin and it means blood putrefaction. Aside from septicemia, sepsis is a medical term defined as “life-threatening organ dysfunction due to a dysregulated host response to infection” (97, 104, 177). Sepsis is a common and lethal syndrome for which no specific treatments exist (105). In the United States, severe sepsis has been shown to occur in about 2% of patients admitted to hospital. The number of cases in the United States exceeds 750,000 per year and was recently reported to be increasing. Whereas the estimated annual economic burden of this condition is about € 2 billion, the lifetime therapeutic management of sepsis is still far from optimal. Sepsis develops when an initial immune response to an endotoxin derived from an infectious agent becomes amplified and deregulated, leading to persistent inflammation and, in the most severe cases, multiorgan failure and death. Sepsis is often thought to result from systemic invasion of the bloodstream by pathogenic organisms. However, several lines of evidence have converged in support of the notion that it also develops in the absence of any invading pathogens as a consequence of tissue injury and/or unrestrained translocation of commensals. Roquilly et al. showed that resolution of the primary infection changed the local lung environment, which led to the development of tolerogenic DCs and macrophages that contributed to immune suppression (178).

As a conceptual framework, we herein propose that sepsis-mediated mononuclear phagocyte system deregulation might occur at the mononuclear phagocyte precursor level. Therefore, strategies aiming to restore the differentiation of DCs, and maintenance of their physiological functions (178) could be beneficial in the treatment of sepsis. However, it remains to be clearly established whether these tolerogenic cells have been biased in their development at the precursor level in a specific environment, such as the kidney. Such a precursor effect is supported by the fact that the impaired capacity of antigen presentation through MHCII molecules lasts for 21 days or more after recovery from the primary infection, which exceeds the short lifetime of DCs. Moreover, if BM precursors are affected by the primary infection, their degree of resilience needs to be measured in the framework of the new paradigm of infection memory (179).

It is worth noting that similar inflammatory pathways that are required for host protection against infectious agents can also be induced in response to sterile tissue damage (180). For instance, type I interferons and TNF cooperatively induce signals to epigenetically reprogram macrophages, thereby rendering them more sensitive to weak signals, such as responses to LPS, while also making them resistant to suppression by IL-10 (131). In other words, the hematopoietic cells integrate infection marks with deleterious consequences and they are then reinitialized differently in a subsequent challenge. It remains to be investigated whether the epigenetic reprogramming of the mononuclear phagocyte system and their precursors may influence long-term disease outcomes. Indeed, new technologies such as single cell RNA sequencing, epigenomic approaches such as ATAC-seq, mass cytometry, and mass histology may improve our knowledge regarding the developmental and functional changes that affect mononuclear phagocytes during various inflammatory conditions such as sepsis and endotoxemia. The availability of different conditional mice to deplete specific genes or subsets would shed light on their specific requirements during sepsis and endotoxemia situations.

Overall, these data indicate functional changes in various populations of myeloid cells over the course of septicemia. However, these results also suggest that sepsis could induce modulations of myeloid cells in terms of the overall populations; promoting the production, survival, differentiation, or proliferation of certain cells at the expense of others. These results therefore suggest that therapeutic strategies aimed at maintaining the number and the functions of the mononuclear phagocyte system, in particular DCs, are likely to limit the immunosuppressive state that is commonly found during septicemia and infectious situations (178).

## Author Contributions

LFP, CL, and MC wrote the manuscript, gave feedback and revised the manuscript.

## Conflict of Interest Statement

The authors declare that the research was conducted in the absence of any commercial or financial relationships that could be construed as a potential conflict of interest.

## Abbreviations

DC(s), dendritic cell(s); cDC, conventional DC; pre-cDC, precursor of cDCs; pDC, plasmacytoid dendritic cell; IFN, interferon; LPS, lipopolysaccharide; MDP, macrophage and DC progenitor; CDP, cDC precursor; cMoP, common monocyte progenitor; MHC-II, major histocompatibility complex class II; BM, bone marrow; Mo-APC, monocyte-derived antigen presenting cells; Mo-DC, monocyte-derived dendritic cell.
